# Progress Toward Rubella Elimination — World Health Organization South-East Asia Region, 2013–2021

**DOI:** 10.15585/mmwr.mm7225a2

**Published:** 2023-06-23

**Authors:** Sudhir Khanal, Sunil Bahl, Lucky Sangal, Deepak Dhongde, Patrick Michael O’Connor, Natasha S. Crowcroft, Michelle Morales

**Affiliations:** ^1^Immunizations and Vaccines Development, World Health Organization South-East Asia Regional Office, New Delhi, India; ^2^Immunization, Vaccines and Biologicals, World Health Organization, Geneva, Switzerland; ^3^Global Immunization Division, Global Health Center, CDC.

During 2013, the 11 countries of the World Health Organization (WHO) South-East Asia Region* (SEAR) adopted the goals of measles elimination and rubella and congenital rubella syndrome (CRS) control^†^ by 2020. During 2019, SEAR countries declared a broader goal for eliminating both measles and rubella^§^ by 2023 (*1*). Before 2013, only five SEAR countries had introduced rubella-containing vaccine (RCV). This report updates a previous report and describes progress toward rubella elimination in SEAR during 2013–2021 (*2*). During 2013–2021, six SEAR countries introduced RCV; all countries in the Region now use RCV in routine immunization. Routine immunization coverage with the first dose of a rubella-containing vaccine (RCV1) increased >600%, from 12% during 2013 to 86% during 2021, and an estimated 515 million persons were vaccinated via RCV supplementary immunization activities (SIAs)^¶^ during 2013–2021. During this time, annual reported rubella incidence declined by 80%, from 5.5 to 1.1 cases per million population. Maldives and Sri Lanka are verified as having achieved rubella elimination; Bhutan, North Korea, and Timor-Leste have halted endemic transmission of rubella virus for >36 months. SEAR has made substantial progress toward rubella elimination; however, intensified measures are needed to achieve elimination. 

Rubella is the leading cause of vaccine-preventable birth defects (*3*). Rubella infection during pregnancy, especially during the first trimester, can result in miscarriage, fetal death, or CRS, a constellation of congenital malformations, frequently including visual, auditory, or cardiac defects. CRS is a cause of mortality among infants and children and a shortened lifespan among adults. Rubella and measles elimination activities are programmatically linked because RCV is administered as a combined measles and rubella vaccine, and rubella cases are detected through case-based surveillance for measles or fever and rash illness (*4*). The WHO SEAR-recommended strategies (*5*) to achieve rubella elimination include 1) achieving and maintaining ≥95% coverage with 2 doses of measles- and rubella-containing vaccine in every district through routine immunization or SIAs; 2) developing and sustaining a sensitive and timely case-based surveillance system for rubella and sentinel site surveillance for CRS that meets recommended performance indicators**; 3) developing and maintaining an accredited laboratory network; 4) achieving timely identification, investigation, and response to rubella outbreaks; and 5) linking with other public health initiatives to achieve the first four strategies. 

## Immunization Activities

RCV1 was introduced in five SEAR countries (Bangladesh, Bhutan, Maldives, Sri Lanka, and Thailand) before 2013 and in the remaining six SEAR countries (Burma [Myanmar],^††^ India, Indonesia, Nepal, North Korea, and Timor-Leste) during 2013–2019. A routine second RCV dose (RCV2) was introduced in three countries (Bhutan, Sri Lanka, and Thailand) before 2013 and in the remaining eight during 2013–2021 (Table 1).

**TABLE 1:** Estimated coverage* with rubella-containing vaccine, recommended age for vaccination, number of confirmed rubella and congenital rubella syndrome cases, and rubella incidence, by country — World Health Organization South-East Asia Region, 2013 and 2021

Country (RCV1, RCV2 introduction)	2013					2021					% Change in rubella incidence 2013–2021
	RCV1 coverage (%)	RCV schedule (age)	No. of confirmed CRS cases	No. of confirmed rubella cases	Rubella incidence^†^	RCV1 coverage (%)	RCV schedule (age)	No. of confirmed CRS cases	No. of confirmed rubella cases	Rubella incidence^†^	
Bangladesh (2012, 2015)	91	9 mos	19	3,034	19.7	97	9 mos, 15 mos	171	129	0.8	−96
Bhutan (2006, 2006)	94	9 mos, 24 mos	0	6	8.2	97	9 mos, 24 mos	1	0	0	−100
Burma (Myanmar)^§^ (2015, 2017)	NA^¶^	NA	NR	23	0.5	44	9 mos,18 mos	NR	3	0.1	−80
India (2017, 2017)	NA^¶^	NA	NR	3,698	2.9	89	9−12 mos, 16–24 mos	NR	1,675	1.2	−59
Indonesia (2017, 2017)	NA^¶^	NA	NR	2,355	9.3	72	9 mos, 18 mos, 7 yrs	229	268	1	−89
Maldives (2007, 2017)	99	9 mos, 18 mos	NR	0	0	99	9 mos, 18 mos	0	0	0	NC
Nepal (2013, 2015)	88	9 mos	NR	755	27.6	90	9 mos, 15 mos	NR	28	0.9	−97
North Korea (2019, 2019)	NA^¶^	NA	0	0	0	NR	9 mos, 15 mos	0	0	0	NC
Sri Lanka (1996, 2001)	99	3 yrs, 13 yrs	4	24	1.1	97	9 mos, 3 yrs	0	0	0	−100
Thailand (1986, 1997)	99	9 mos, P1**	0	539	7.7	96	9 mos, 1.5 yrs	NR	NR	NR	—^††^
Timor-Leste (2016)^§§^	NA^¶^	NA	NR	0	0	79	9 mos, 18 mos	0	0	0	NC
**Total**	**12** ^¶¶^	**—**	**23**	**10,434**	**5.5**	**86^¶¶^**	**—**	**401**	**2,103**	**1.1**	−**80**

**Abbreviations:** NA = not applicable; NC = not calculated; NR = not reported during the year; P = primary grade of school; RCV = rubella-containing vaccine; RCV1 = first dose of RCV; RCV2 = second dose of RCV; SEAR = South-East Asia Region; WHO = World Health Organization.

* WHO-UNICEF coverage estimates, 2021 revision (as of July 2022). https://immunizationdata.who.int/

^†^ Cases per 1 million population.

^§^ *MMWR* uses the U.S. Department of State’s short-form name “Burma”; WHO uses “Myanmar.”

^¶^ Dose was not included in the vaccination schedule for that year.

** Given to primary grade 1 students (aged approximately 7 years).

^††^ Change in rubella incidence could not be calculated because cases were not reported via WHO-UNICEF Joint Reporting Form in 2021.

^§§^ RCV1 and RCV2 were introduced during 2016.

^¶¶^ The regional estimates are calculated as part of WHO-UNICEF coverage estimates, in which the denominator is the total birth cohort of the region irrespective of the reporting status, and the numerator is the sum of estimated vaccinated children and adolescents from all reporting countries.

WHO and UNICEF estimated that regional RCV1 coverage increased from 12% during 2013 to 86% during 2021 (*6*) (Figure); five countries reported ≥95% RCV1 coverage during 2021 (Table 1). The highest regional RCV1 coverage (93%) was achieved during 2019, just before the start of the COVID-19 pandemic. During 2013–2021, SIAs with RCV were conducted in 10 SEAR countries (all except Sri Lanka) and reached more than 514 million persons.^§§^

**FIGURE:**
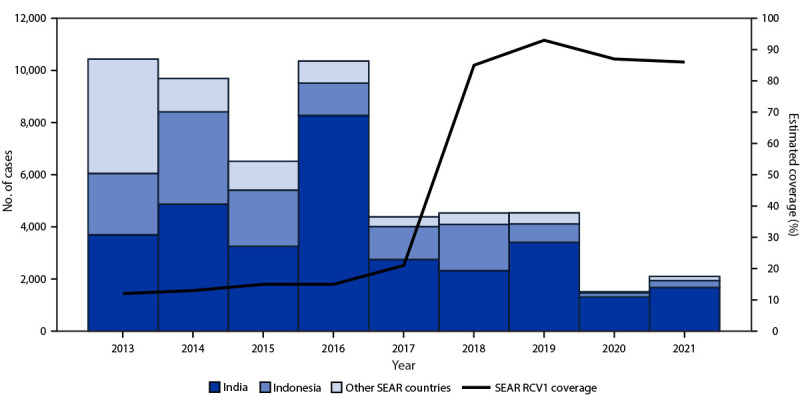
Number of reported rubella cases,* by country,**^†,§^** and estimated first dose rubella-containing vaccination coverage**^¶ ^**— World Health Organization South-East Asia Region, 2013–2021 Source: https://immunizationdata.who.int/ **Abbreviations:** RCV = rubella-containing vaccine; SEAR = South-East Asia Region; WHO = World Health Organization. * Cases of rubella reported to WHO and UNICEF through the Joint Reporting Form to the WHO Regional Office for SEAR. ^†^ Other countries in the region include Bangladesh, Bhutan, Burma (Myanmar), Maldives, Nepal, North Korea, Sri Lanka, Thailand, and Timor-Leste. ^§^ *MMWR* uses the U.S. Department of State’s short-form name “Burma”; WHO uses “Myanmar.” ^¶^ Data are from WHO and UNICEF estimates of routine first RCV dose coverage for SEAR.

## Surveillance Activities

By 2021, case-based measles and rubella surveillance with laboratory confirmation of suspected cases^¶¶^ was implemented in all countries in the region. As an integral component of the WHO Global Measles and Rubella Laboratory Network, a measles-rubella laboratory network was established in the region by 2003, and by 2021, the regional laboratory network included 58 proficient laboratories*** with one regional reference laboratory in Thailand; all countries had at least one proficient laboratory. During 2013, two of the 11 countries achieved the sensitivity indicator target of two or more discarded nonmeasles, nonrubella cases per 100,000 population, and the regional discarded case rate was 0.91; this increased to 1.52 during 2021. However, during 2021, only five countries achieved the target discarded case rate of two or more per 100,000 population (Table 2).

**TABLE 2:** Year of initiation of surveillance for rubella and key surveillance performance indicator of nonmeasles, nonrubella discard rate, by country and year — World Health Organization South-East Asia Region, 2013–2021

Country	Year rubella surveillance activities initiated			Discarded nonmeasles, nonrubella reporting rate*	
	Rubella^†^	Fever and rash^§^	CRS^¶^	2013	2021
Bangladesh	2008	2021	2012	1.1	2.00
Bhutan	2007	2015	2015	12.9	19.44
Burma (Myanmar)**	2008	2019	2016	0.34	0.03
India	2005	2019	2016	1.51	1.69
Indonesia	2008	2019	2014	0.54	0.69
Maldives	2014	2017	2015	0	4.21
Nepal	2007	2019	2014	0.90	9.97
North Korea	2006	2018	2015	0.26	1.60
Sri Lanka	2004	2015	1991	2.99	0.10
Thailand^††^	1973	2018	1973	0.63	0.30
Timor-Leste	2009	2018	2016	0	2.43
**Total**	**NA**	**NA**	**NA**	**0.91**	**1.52**

**Source:** https://www.who.int/publications/i/item/SEAR-MR-Bulletin-Q3-2021

**Abbreviations:** CRS = congenital rubella syndrome; NA = not applicable; SEAR = South-East Asia Region; WHO = World Health Organization.

* Discarded cases per 100,000 population. A discarded case is defined as a suspected case that has been investigated and determined to be neither measles nor rubella using 1) laboratory testing in a proficient laboratory or 2) epidemiologic linkage to a laboratory-confirmed outbreak of another communicable disease that is not measles or rubella. The discarded case rate is used to measure the sensitivity of measles-rubella surveillance.

^†^ The year any form of CRS was initiated in the country. Countries defined a suspected measles/rubella case as “acute fever with maculopapular rash and at least one of the following: cough, coryza, or conjunctivitis.”

^§^ The year laboratory supported case-based surveillance with definition of a suspected measles/rubella case as “acute fever with maculopapular rash” was initiated in the country.

^¶^ The year any form of CRS surveillance was initiated in the country at national level.

** *MMWR* uses the U.S. Department of State’s short-form name “Burma”; WHO uses “Myanmar.”

^††^ CRS surveillance was initiated during 1973. At that time, the same reporting code was used for both rubella and CRS; however, during 2020, CRS was formally identified with its own reporting code separate from rubella.

All countries in SEAR have established CRS surveillance. North Korea, Sri Lanka, and Thailand report CRS cases as part of their national integrated disease surveillance programs. The remaining eight countries identify CRS cases through sentinel site surveillance. The number of SEAR countries reporting CRS cases through the WHO-UNICEF Joint Reporting Form increased from six during 2013 to seven during 2021 (Table 1).

## Rubella and CRS Incidence and Rubella Virus Genotypes

During 2013–2021, the number of reported^†††^ rubella cases in the region decreased by 80%, from 10,434 to 2,103 (Figure). Annual rubella incidence also declined by 80%, from 5.5 to 1.1 cases per 1 million population (Table 1). The number of reported CRS cases increased from 23 to 401, likely because of establishment or enhancement of CRS surveillance in multiple SEAR countries.

During 2013–2021, rubella virus genotypes detected in patient isolates in the region included 2B in India and Thailand, with endemic 1E in Thailand, and 1J in India. However, the number of specimens collected and tested for genotyping was low, limiting interpretation about transmission.

## Regional Verification of Rubella Control and Elimination

The WHO South-East Asia Regional Verification Commission (RVC) for measles and rubella elimination was established during 2016 and developed an updated framework for verification of measles and rubella elimination during 2020 (*7*). National verification committees were established in all 11 countries, providing annual reports on progress toward measles and rubella elimination to the RVC. As of 2021, the RVC has verified rubella elimination in Maldives (2020) and Sri Lanka (2020). In addition, three countries (Bhutan, North Korea, and Timor-Leste) have halted endemic transmission of rubella for >36 months and were awaiting verification of elimination (*8*).

## Discussion

During 2013–2021, substantial progress was made toward rubella elimination in WHO SEAR. Through the implementation of the regional strategies, estimated RCV1 coverage increased by >600%, and reported rubella incidence declined by 80%. The increase in the number of reported CRS cases during 2013–2021 likely reflects improved surveillance in the countries that initiated CRS surveillance after 2013, rather than an increase in rubella among susceptible pregnant women and CRS in their infants (*3*). By the end of 2021, two of the 11 countries had been verified as having eliminated endemic rubella transmission. As of May 2023, an additional three countries with interrupted rubella virus transmission for >36 months are awaiting verification of elimination.

Despite these successes, challenges to achieving rubella elimination in SEAR exist. During the COVID-19 pandemic, routine RCV1 coverage in the region declined from 93% during 2019 to 86% during 2021. During 2021, among the estimated 25 million infants who did not receive RCV1 worldwide, approximately 18% lived in SEAR, including 2.4 million in India and 1.2 million in Indonesia (*9*). In addition, rubella surveillance activities were affected by the pandemic, likely related to COVID-19 mitigation measures (e.g., physical distancing or masking) that decreased transmission of rubella and other respiratory viruses, in addition to declines in clinic visits for febrile rash illness because of movement restrictions imposed nationally, and the deployment of surveillance personnel to pandemic response activities. A recent independent review of progress toward measles and rubella elimination in SEAR concluded that several challenges, including immunity gaps, suboptimal surveillance sensitivity, inadequate outbreak response and preparedness, funding gaps, and the negative effects of the COVID-19 pandemic on immunization programs threatened the achievement of the 2023 target (*10*).

The findings in this report are subject to at least three limitations. First, coverage estimates are based on administrative data and might be inaccurate because of errors in recording doses administered or in estimates of the target populations. Second, surveillance data might underestimate true disease incidence because not all rubella infections cause fever, not all patients seek care, and not all rubella cases in patients who seek care are reported. In addition, not all countries are consistently reporting CRS cases through the Joint Reporting Form. Finally, genotype data are based on a limited and nonrepresentative number of sequences and do not necessarily reflect the predominant genotypes in the region.

Achieving rubella elimination in WHO-SEAR by 2023 will require urgent, intensified measures by countries to implement strategies in a very short time. The resetting of a new target date represents an opportunity to galvanize activities and maintain momentum in the region to 1) obtain the highest level of national commitment from SEAR countries and support from partners; 2) strengthen routine immunization and achieve ≥95% coverage with RCV1; 3) conduct high-quality SIAs; 4) enhance surveillance sensitivity and increase collection of specimens for rubella virus detection and genotyping; and 5) leverage elimination activities to enhance measures to restore routine immunization services and reduce immunity gaps for all vaccine-preventable diseases. With the regional birth cohort representing 24% of the world’s infants surviving beyond age 1 year, progress toward rubella elimination in SEAR represents an important opportunity to decrease rubella-related death, disability, and illness worldwide.

**Summary**

**What is already known about this topic?**

During 2013, coverage with the first dose of rubella-containing vaccine (RCV1) in the World Health Organization South-East Asia Region was 12%, and only five countries in the region had introduced RCV into their routine immunization programs.

**What is added by this report?**

By 2021, all 11 SEAR countries had introduced RCV1, and estimated regional RCV1 coverage increased from 12% to 86%; rubella incidence declined by 80%. Maldives and Sri Lanka achieved rubella elimination; Bhutan, North Korea, and Timor-Leste have halted endemic transmission of rubella virus for >36 months.

**What are the implications for public health practice?**

SEAR has made substantial progress toward rubella elimination. To achieve regional rubella elimination by 2023, optimal and accelerated measures to implement all elimination strategies are needed.

## Footnotes

* Bangladesh, Bhutan, Burma (Myanmar), India, Indonesia, Maldives, Nepal, North Korea, Sri Lanka, Thailand, and Timor-Leste.

† Measles elimination is defined as the absence of endemic measles cases for a period of ≥12 months in the presence of adequate surveillance. Rubella and CRS control is defined as a 95% reduction in disease incidence from the 2013 level.

§ Rubella elimination is defined as the absence of endemic rubella cases for a period of ≥12 months in the presence of adequate surveillance.

¶ Generally, SIAs for measles and rubella are carried out using two target age ranges. An initial, nationwide catch-up SIA focuses on all children and adolescents aged 9 months–14 years, with the goal of eliminating susceptibility to measles and rubella in the general population. Generally, follow-up SIAs are conducted nationwide every 2–4 years and target children aged 9–59 months with the goal of eliminating any measles and rubella susceptibility that has accumulated in recent birth cohorts and protecting children who did not respond to the first measles vaccination.

** These indicators include 1) two or more discarded nonmeasles, nonrubella cases per 100,000 population at the national level per year (a suspected case that has been investigated and determined to be neither measles nor rubella using laboratory testing in a proficient laboratory or epidemiologic linkage to a laboratory-confirmed outbreak of another communicable nonmeasles, nonrubella disease), to measure surveillance sensitivity; 2) two or more discarded nonmeasles, nonrubella cases per 100,000 population per year in ≥80% of subnational administrative units; 3) testing of ≥80% of suspected measles cases for measles immunoglobulin M antibodies; 4) adequate investigation conducted within 48 hours of notification of ≥80% of suspected cases; 5) adequate collection of samples for detecting measles or rubella viruses and testing in accredited laboratory of ≥80% of laboratory-confirmed transmission chains; and 6) an annualized incidence of zero confirmed endemic measles cases.

†† *MMWR* uses the U.S. Department of State’s short-form name “Burma”; WHO uses “Myanmar.”

§§
https://immunizationdata.who.int/

¶¶ The definition of a suspected measles or rubella case was “acute fever with maculopapular rash” in all SEAR countries.

*** A laboratory that has met defined criteria as outlined at https://www.who.int/publications/i/item/framework-for-verifying-elimination-of-measles-and-rubella

††† Countries report the number of rubella or CRS cases annually using the WHO-UNICEF Joint Reporting Form.
